# Development of a scoring parameter to characterize data quality of centroids in high-resolution mass spectra

**DOI:** 10.1007/s00216-022-04224-y

**Published:** 2022-07-25

**Authors:** Max Reuschenbach, Lotta L. Hohrenk-Danzouma, Torsten C. Schmidt, Gerrit Renner

**Affiliations:** 1 Instrumental Analytical Chemistry, University of Duisburg-Essen, Universitätsstr. 5, 45141 Essen, Germany; 2 Centre for Water and Environmental Research (ZWU), University of Duisburg-Essen, Universitätsstr. 2, 45141 Essen, Germany; 3 IWW Water Centre, Moritzstr. 26, 45476 Mülheim an der Ruhr, Germany

**Keywords:** Data processing, HRMS, Centroiding, Data quality

## Abstract

High-resolution mass spectrometry is widely used in many research fields allowing for accurate mass determinations. In this context, it is pretty standard that high-resolution profile mode mass spectra are reduced to centroided data, which many data processing routines rely on for further evaluation. Yet information on the peak profile quality is not conserved in those approaches; i.e., describing results reliability is almost impossible. Therefore, we overcome this limitation by developing a new statistical parameter called data quality score (DQS). For the DQS calculations, we performed a very fast and robust regression analysis of the individual high-resolution peak profiles and considered error propagation to estimate the uncertainties of the regression coefficients. We successfully validated the new algorithm with the vendor-specific algorithm implemented in *Proteowizard’s msConvert*. Moreover, we show that the DQS is a sum parameter associated with centroid accuracy and precision. We also demonstrate the benefit of the new algorithm in nontarget screenings as the DQS prioritizes signals that are not influenced by non-resolved isobaric ions or isotopic fine structures. The algorithm is implemented in Python, R, and Julia programming languages and supports multi- and cross-platform downstream data handling.

**Supplementary Information:**

The online version contains supplementary material available at 10.1007/s00216-022-04224-y.

## Introduction

High-resolution mass spectrometry (HRMS) is an established method in many research areas, such as metabolomics, forensics, and environmental analysis, due to its ability to determine the accurate molecular mass [1, 2]. There exist diverse HRMS techniques, and the most common ones are the time-of-flight mass spectrometer (TOF–MS), Orbitrap mass spectrometer, and Fourier transform ion cyclotron resonance mass spectrometer (FT-ICR-MS) [3–5]. HRMS can be coupled with chromatographic methods, e.g., high-performance liquid chromatography (HPLC), to first separate and then analyze complex substance mixtures [6]. Besides conventional target analysis aiming for quantification, HRMS is applied in the area of suspect and non-target screening to gather qualitative information used for structure elucidation and identification of unknown analytes [7–9]. In this context, estimations of the accurate masses and isotopic patterns are important elements for structure elucidation [10].

Mass spectra are stored in either profile or centroid mode; the mass peak profiles possess positions, widths, and intensity distributions, while centroid peaks are pairs of position and intensity [11]. Peak profiles in high-resolution mass spectrometry follow a certain peak shape function. This function describes how the signal intensities are distributed around a central value. Orbitrap-MS peak profiles are generated through Fourier transform by combining absorption and magnitude mode spectra, which are subjected to smoothing and filtering [12]. The peak shape function for Orbitrap-MS is considered symmetric following *Lorentzian* or *Gaussian* functions, but in detail depends on multiple factors, such as the steps applied in the construction of the peaks (apodization, zero-filling) [12–14]. Centroid positions coincide with local maxima of symmetric peak profiles. Deviations from symmetry can occur when overlaps are present, such as isobaric interferences or isotopic fine structures [15, 16].

The processing of centroided datasets is significantly less time demanding, whereas profile mass spectra have higher information content. However, a prerequisite for many established data processing routines is that the mass spectra are present in centroid mode [17, 18]. There exist centroiding tools such as *msConvert*, which is part of the software package *Proteowizard* and is widely applied for converting peak profiles to centroid data [19]. Some software appends additional information on the former mass resolution and the signal-to-noise ratio of centroids [20]. Furthermore, with applications such as *RawTools*, instrument-specific raw files can be read and processed using the implemented vendor libraries [21]. Although tools such as *msConvert* and *RawTools* with their implemented algorithms can be used by the public, operational details on the vendor-specific algorithms used for centroidization are not published.

Different approaches exist to estimate the centroid positions in high-resolution profile mass spectra. Boulet et al. (2021) used the first derivative of the *Savitzky-Golay* filter [22]. Sanchez Brotons et al. (2021) applied a 2D *Gaussian* kernel smoothing on profile mass spectra within their LC–MS/MS preprocessing toolset to describe the centroid position, also considering the chromatographic time domain [14]. Recently, Samanipour et al. (2021) developed a centroiding workflow that can estimate resolution-based mass peak widths using a self-adjusting algorithm and an initial peak width guess [23].

Various tools are available for processing HRMS data, which differ considerably in their results [24]. A problem in centroid processing that has received little attention so far is that meaningful information about the original quality of the data is usually lost. In centroiding, mass peaks that are subject to a particular distribution are represented by an individual *m*/*z*-intensity-value pair. As peak profiles are often not entirely resolved, not every centroid is generated based on consistent data quality [25]. The peaks differ, for instance, by having a different number of data points, and overlaps of peaks without complete separation (e.g., isobaric ions or isotopic fine structures) occur. However, the qualities of the peak profiles are not apparent using the previously established procedures, as the exported centroids do not conserve the information: for example, whether they are originating from monoisotopic peaks. Therefore, it is no longer possible to analyze if the reliabilities of the generated centroids differ.

Our study aims to combine the best of both modes: profile and centroid. Therefore, we overcome the limitations of the conventional centroiding routines: achieving data reduction while preserving the relevant information on former peak width and data quality. To that end, we develop a fast centroiding algorithm that quantifies the peak quality in a Data Quality Score (DQS). The DQS will be attached to each centroid in the exported centroid list. This shall increase the transparency of the centroiding process concerning the reliability of the former peak profiles.

## Material and methods

### Sampling and sample preparation

Exemplary samples from a non-target screening approach have been prepared and measured with HPLC-HRMS for centroiding algorithm development and validation. The samples measured were grab samples taken from a wastewater treatment plant effluent in Warburg (Stadtwerke Warburg GmbH, Warburg, Germany). The effluent samples usually contain a high load of organic substances, which increases matrix-associated effects in HRMS such as signal overlay. Therefore, they serve as suitable datasets to test the algorithm’s robustness. Detailed instructions on sampling and sample preparation were published by Hohrenk et al. (2020) [24].

### Instrumental analysis

The HPLC system Dionex UltiMate 3000 (Thermo Scientific, Bremen, Germany) was used for chromatographic separation [26]. Details of the chromatography can be obtained from the SI and elsewhere [24]. The HPLC was coupled with a Q Exactive Orbitrap mass spectrometer (Thermo Scientific, Bremen, Germany) for accurate mass detection. The Orbitrap measurements were performed as full scan mode in MS1 (*m*/*z* 100–1000) with a mass resolution of 70,000 at *m*/*z 200* and data-dependent MS2 scans with the five most intense peaks per mass spectrum at a mass resolution of 17,500 at *m/z* 200. The algorithm was developed for MS1 and MS2 spectra and can be applied for ESI-positive and ESI-negative measurements. All examples shown within this work refer to MS1 spectra in ESI-positive mode. However, for algorithm development, the chromatographic domain of the datasets was neglected, as the qualities of consecutive mass spectra are assumed to be independent. Therefore, the developed centroiding algorithm can be used universally for either measurement with or without prior chromatographic separation.

### Data handling

The instrumental measurement raw files (in case of Orbitrap-MS, Thermo *.raw format) were converted to profile mass spectra *.mzXML with *Proteowizard’s msConvert* to import them more easily into the applied programming environments [19]. However, *msConvert* was not used for centroiding in this step, but only for conversion to open format. The new centroiding algorithm is platform-independent and implemented and available in the programming languages Python (v3.9), R (v4.0.4), and Julia (v1.6.3). Within the programming environment, third-party packages have been applied, listed in the supplementary material (Table S3). The third-party packages are installed automatically in our ready-to-use application, which lowers the initial hurdle to work with our algorithm. Finally, the centroided data are exported to *.csv at the end of the processing workflow.

### Regression workflow for peak profile centroiding

Centroiding reduces multiple data points that form a peak profile to a single *m*/*z*-intensity pair that estimates its theoretical mode. The core idea of our new method is a regression of the measured profile data with an adequate model. In this subsection, we present the concept of Data Quality Score for *Gaussian*-shaped peak profiles commonly observed in Orbitrap-MS as the results presented and discussed focus on this instrument [14]. TOF–MS can generate peak profiles that are non-symmetric and thus cannot be appropriately described by a *Gaussian* model [15]. Principally, different asymmetric models are available for this purpose; however, many can only be accessed by non-linear regression [27]. In this work, we present the following approach as a proof of concept: by switching from the *Gaussian* to *Bi-Gaussian* model, profile mass spectra generated by TOF–MS can be analyzed. The central concept of the peak regression presented in this study is not changed due to a different peak model. Therefore, details on the TOF–MS centroiding routine can be found in a specific section in the supplementary material. The development of the algorithm for TOF–MS was performed using a dataset by Samanipour et al. (2021) [23]. The *Gaussian* peak function we use for this purpose will be derived within this paper as well. The model parameters include all relevant peak information, such as position, height, and width, and associated uncertainties. These uncertainties are essential for the subsequent scoring of the centroids. For regression, we used *Caruana*’s approach, which allows linearizing the non-linear *Gaussian* curve fitting to minimize calculation efforts [26]. However, the utilized regression can only be applied to a single peak profile at a time. Therefore, the mass spectra must first be divided into smaller packages containing isolated peak profiles. In Orbitrap-MS, the resolved peak profile intensities are surrounded by zeros. This circumstance is helpful as these zeros can be considered predefined peak boundaries.

**Fig. 1:**
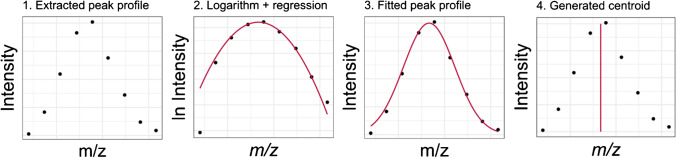
Procedure of our new algorithm for centroiding profile mass spectra: The standard errors associated with the parabola in step 2 are used for the calculation of the Data Quality Scores

However, overlapping peak profiles also exist, which will not work without splitting in advance [26]. In this context, valley points indicate splitting locations, assigning the valley point itself to both profiles. Potentially, deconvolution can be used to determine the centroids of non-resolved overlapping peaks. We deliberately decided against deconvolution. This would lead to non-linear regression problems that are very time demanding, usually not purposeful due to the big data files generated, for example, by HPLC-HRMS. When no deconvolution is applied, we neglect that neighboring peaks influence each other in their shapes through an overlap in their peak area. However, the accuracy benefit of deconvolution is limited for peaks with optimal nearly *Gaussian* shapes. A schematic overview of how the centroids are generated from the profiles is shown in Fig. 1.

After isolating all the peak profiles, the regression itself can be performed using the already mentioned *Gaussian* model as the basis function. Thus, the function that describes the intensity curve is given by:

1 $$\widehat{I}(x) =\widehat{I}_0 \cdot \exp \left(-\frac{(x-\widehat{x}_0)^2}{2\widehat{\sigma}^2}\right)$$

where $\widehat{I}\left(x\right)$ is the estimated intensity at the independent variable $x$, ${\widehat{I}}_{0}$ is the estimated peak height, and ${\widehat{x}}_{0}$ is the estimated peak position which equals the centroid in our case. The estimated standard deviation $\widehat{\sigma }$ of the *Gaussian* peak is a measure for peak width.

Following *Caruana*, the given model (Eq. 1) is not directly accessible with linear regression which would lead to a time-consuming iterative solving process and is therefore not suitable for the millions of peak profiles HRMS data can contain. To that end, we perform log-transformation of Eq. 1 to estimate peak parameters and their uncertainties with a second-order linear regression without the need for iterative optimization. As shown by Caruana et al. (1986), the linearized model is described by [26]:

2 $$\underbrace{\ln \widehat{I}(x)}_{\hat{y}} = \underbrace{\left(\ln \widehat{I}_0 - \frac{\widehat{x}_0^2}{2\widehat{\sigma}^2}\right)}_{\widehat{\beta}_0} + \underbrace{\left(\frac{\widehat{x}_0}{\widehat{\sigma}^2} x\right)}_{\widehat{\beta}_1x} - \underbrace{\left(\frac{1}{2 \widehat{\sigma}^2} x^2\right)}_{\widehat{\beta}_2x^2}$$

which is a second-order polynomial, and the parameters of the *Gaussian* peak are estimated via [26]:

3 $$\widehat{x}_0 = -\frac{\widehat{\beta}_1}{2\widehat{\beta_2}}$$

4 $$\widehat{\sigma} = \sqrt{ -\frac{1}{2\widehat{\beta}_2}}$$

5 $$\widehat{I}_0 = \exp \left( \widehat{\beta}_0 - \frac{\widehat{\beta}_1^2}{4\widehat{\beta}_2} \right)$$

The estimated *Gaussian* peak area $\widehat{A}$ can be obtained by:

6 $$\widehat{A} =\widehat{I}_{0} \cdot \widehat{\sigma} \cdot \sqrt{2\pi}$$

The linear regression problem (Eq. 2) is parallelized and solved using matrix algebra. Due to the log-transform, noise strongly influences the fit results, especially with an increased distance to the centroid position. However, weighted linear regression will treat this problem [28]. The weights can be calculated as follows:

7 $${w}_{i}={\left(\frac{{I}_{i}}{\sum {I}_{i}}\right)}^{2}$$

with ${w}_{i}$ being the weight of data point $i$ and ${I}_{i}$ is the $i$ th intensity. The weight with a power of 2 fits very well in the case of Orbitrap-MS data but could potentially be changed by the user. The regression parameters $\widehat{\beta }$ are estimated by:

8 $$\widehat{\beta}=(\varvec{X}^T \varvec{W} \varvec{X})^{-1} \varvec{X}^T \varvec{W} \varvec{Y}=\left(\begin{array}{c}\widehat{\beta}_0\\\widehat{\beta}_1\\\widehat{\beta}_2\\\end{array}\right)$$

where ${\varvec{X}}$ is the Vandermonde matrix, ${\varvec{Y}}$ is the intensity matrix, and ${\varvec{W}}$ is the diagonal matrix containing the weights. Sparse matrices are applied here for fast calculation with low memory demands. For one spectrum, the sparse matrix has the dimension $n \times m$ with $n$ the number of data points in that spectrum and $m$ being the number of peak profiles. For the second-order linear regression, the sparse Vandermonde matrix ${\varvec{X}}$ for two exemplary peak profiles consisting of $i$ and $j$ data points is given by:

9 $${\varvec{X}}=\left(\begin{array}{cccccc}1& {\mathrm{x}}_{11}& {\mathrm{x}}_{11}^{2}& *& *& *\\ 1& {\mathrm{x}}_{12}& {\mathrm{x}}_{12}^{2}& *& *& *\\ \vdots & \vdots & \vdots & *& *& *\\ 1& {\mathrm{x}}_{1\mathrm{i}}& {\mathrm{x}}_{1\mathrm{i}}^{2}& *& *& *\\ *& *& *& 1& {\mathrm{x}}_{21}& {\mathrm{x}}_{21}^{2}\\ *& *& *& 1& {\mathrm{x}}_{22}& {\mathrm{x}}_{22}^{2}\\ *& *& *& \vdots & \vdots & \vdots \\ *& *& *& 1& {\mathrm{x}}_{2\mathrm{j}}& {\mathrm{x}}_{2\mathrm{j}}^{2}\end{array}\right)$$

where asterisks are zeros and do not occupy memory in sparse matrix notation.

### Derivation of the data quality score

In the following, the DQS for HRMS will be derived. A high DQS shall describe a peak profile of high quality, and therefore high accordance with a *Gaussian* peak. The *Gaussian* peak shape depends on three parameters height, width, and position, which are derived from the three regression coefficients (see Eq. 8). Accordingly, if one of these regression coefficients could only be estimated with low confidence (expressed through a high standard error), the data quality is reduced with respect to the *Gaussian* model. Therefore, we need a measure that incorporates all three regression coefficients, and their associated standard errors, and that is independent of the scale of the *Gaussian* peak to estimate the overall peak quality. Alternatively, Bayesian statistics could estimate the regressions’ uncertainties. However, this is significantly more computationally expensive due to the Monte Carlo method involved and thus not feasible for us, since a sample of *n* = 1000 did not provide any significant variation in the results (SI). To that end, we propose using the relative error of the *Gaussian* peak area for DQS calculations:

10 $$\text{DQS} =1 - \text{erf} \left(\frac{\Delta\widehat{A}}{\widehat{A}}\right)$$

where ${\text{erf}}\left(x\right)$ is the *Gaussian* error function and $\Delta \widehat{\mathrm{A}}$ can be deduced from error propagation:

11 $$\Delta\widehat{A} = \sqrt{ \left( \frac{\partial\widehat{A}}{\partial\widehat{I}_0 }\right)^2 \Delta\widehat{I}_0^2 + \left(\frac{\partial\widehat{A}}{\partial \widehat{\sigma} }\right)^2 \Delta \widehat{\sigma}^2 }$$

$\Delta \widehat{A}$ and $\Delta \widehat{\sigma}$ are calculated from the regression standard errors by applying the rules of error propagation for Eqs. 4 and 5. A plot of the function (Eq. 10) used to obtain the DQS is given in the supplementary material in Figure S2. In Eq. 10, the *Gaussian* error function was applied to calculate the DQS because the resulting equation always outputs values between 0 and 1. A relative error of 0 leads to a DQS of 1, while an infinite relative error leads to a DQS of 0.

The regression standard errors are estimated through the variance–covariance matrix of the regression coefficients:

12 $$\bf{\widehat{s}^2}(\bf{\beta})=\mathit{MSE} \cdot (\bf{X}^T\bf{W}\bf{X})^{-1}$$

With the mean square error MSE of regression calculated by:

13 $$\mathit{MSE} = \frac{1}{n-3} \sum w_i (y_i - \widehat{y}_i)^2$$

With $n-3$ equal to the degrees of freedom, ${y}_{i}$ is the logarithmized raw intensity, and $\widehat{{y}_{i}}$ is the fitted logarithmized intensity.

In some cases, the peak fit does not follow the expected *Gaussian* shape. Peaks that do not fulfill the following criteria are removed:

1. Coefficients ${\widehat{\beta }}_{2}<0$ as otherwise *Gaussian*’s standard deviation $\widehat{\sigma }$ is not defined
2. At least 4 data points to obtain a fit with a residual degree of freedom

Criterion 1 requires that all second-order linear regressions show downward-opening parabolas in the logarithmic domain. Otherwise, the determination of $\widehat{\sigma }$ in Eq. 4 is only valid for complex numbers, which makes no sense from an analytical point of view.

## Results and discussion

### Data quality score of centroids

The calculated DQS values from the real water sample measured with HPLC-Orbitrap-MS are presented and discussed in this section. The DQS describes the individual qualities of the peak profiles. High values show a correspondingly high agreement with the chosen *Gaussian* model and can thus be considered monoisotopic. In a broader perspective, the average quality or dependencies of the data quality on, e.g., *m*/*z*, can be determined.

On average, the regressions have a low normalized root mean square error (nRMSE) value of 0.40%. The nRMSE is calculated with the square root of MSE (Eq. 13) and normalized with the mean logarithmic intensity over the peak profiles. Accordingly, the residuals show only a small spread, indicating high regression accuracy; i.e., the regression model is suitable to describe the peaks and their characteristic elements: position, width, and height. The *Gaussian* model has already been applied for regression of Orbitrap-MS peak profiles [14]. However, it is mentioned in the literature that the obtained peak profiles for Orbitrap-MS can follow a *Lorentzian* function. For this case, the regression model would have to be adapted, but the consideration to determine the data quality using the error propagation is generally applicable there as well [12, 13]. The peak profiles consist of a relatively small number of data points (4–20, mean 6.9).

**Fig. 2:**
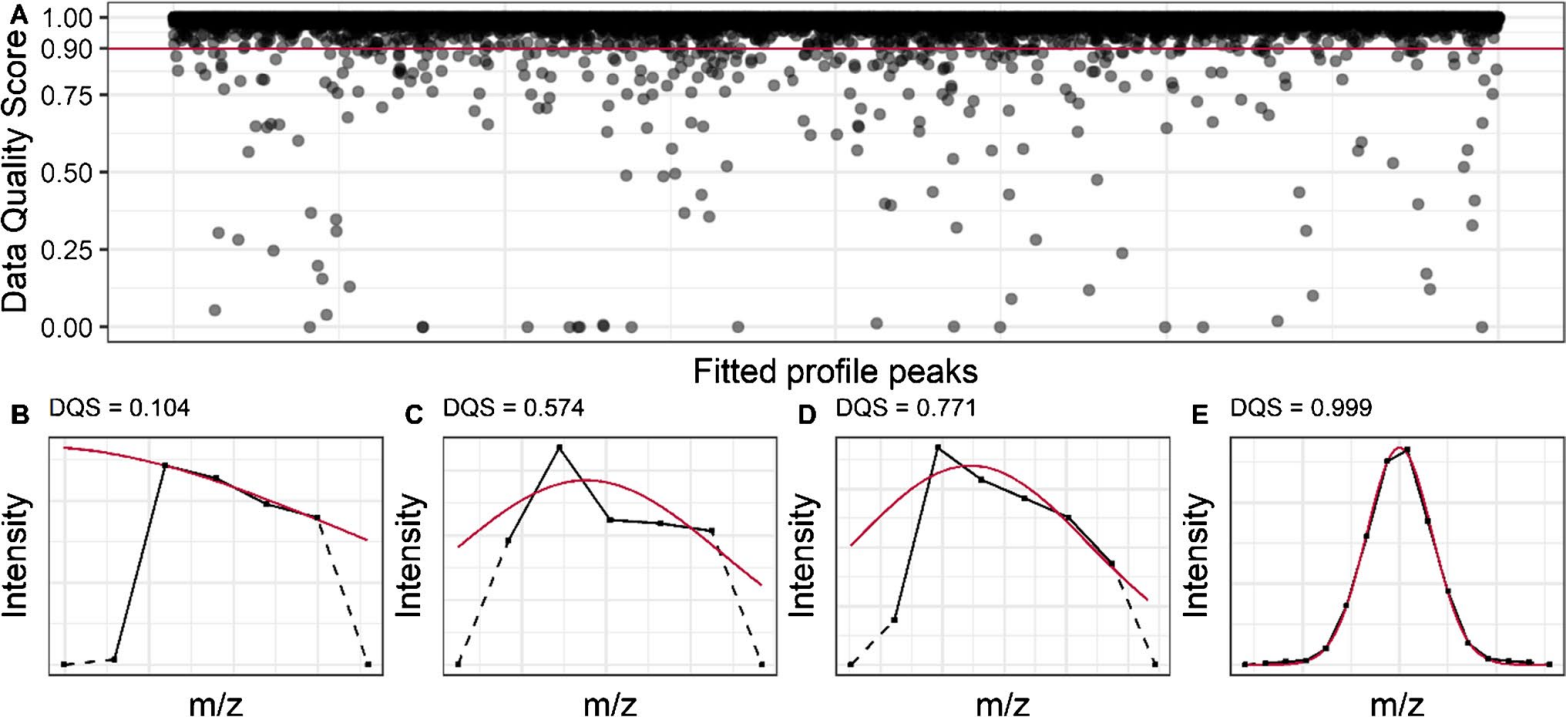
**A** Scatterplot of Data Quality Scores (DQS). Most peak profiles (99.7%) obtain a DQS above 0.90 which means that they show good agreement with the *Gaussian* model. **B**–**E** Four examples for peak profiles with fits (red) with different Data Quality Scores. The dotted lines mark the connection to the equal zero intensities located around the peak profiles. These values are not included for fitting

Figure 2A demonstrates the distribution of the DQS for peak profiles. At least 99.7% of the centroids’ DQS exceed 0.90, and 95.1% even exceed 0.99. In the case of low DQS, several causes for deviation from the ideal shape can occur. There are cases like in Fig. 2C where there is no clear presence of a monomodal peak within the profile. Furthermore, peaks with a high asymmetry, or that seem to be cut off at one side, can be detected (Fig. 2B and D). An increased mass resolution (and thus, more data points per peak) could reduce the number of these cases as peak shoulders should be better resolved. If the mass resolution is decreased, e.g., through measurement in the higher *m*/*z* range in Orbitrap-MS, lower DQS values are obtained as the regression coefficient standard errors depend on the statistical degrees of freedom. As shown in Fig. 2E for scores close to 1, the *Gaussian* fit describes the peaks very well. The higher the score, the better the fit describes the position and height of the profile peak; and thus, the DQS is proportional to the reliability of the received centroids. Although the peaks consist, on average, of only about 7 data points, the agreement with the *Gaussian* model is almost perfect in many cases. A *t*-test reveals that there is no significant correlation between peak intensity and DQS in Orbitrap-MS (*r* = 0.0157, *p* = 0.299, *n* = 1129). Thus, high peak intensity does not automatically imply a high peak quality for Orbitrap-MS. In contrast, for TOF–MS data, a significant correlation exists (*r* = 0.0822, *p* = 0.0043, *n* = 1200). Therefore, an absence of correlation in Orbitrap-MS could be due to pre-processing and the apodization step to generate the peak profiles. Centroids’ quality depends on the measurement conditions and the investigated analytes. Especially in measurements at high masses, non-separated isotopic fine structures or isobaric substances occur due to limited mass resolution. However, DQS can indicate if a centroid originates from a monoisotopic profile peak, which is of fundamental importance for the mass assignment and elemental composition. This is supported by a simulation analyzing the influence of peak-to-peak resolution (in multiples of peak width σ) on the resulting DQS that can be found in the supplementary material (Fig. S5 and Fig. S6). Therefore, DQS could serve as an alternative criterion to intensity thresholds to filter out centroids originating from non-monoisotopic peak profiles. If Orbitrap peak profiles are apodized differently and, therefore, follow, e.g., a *Lorentzian* shape, our algorithm would still be able to detect strong asymmetries with low DQS values. However, as the regression standard errors increase, the DQS distribution should be shifted to lower scores than the *Gaussian*-shaped peak profiles. In this context, further models, e.g., *Lorentzian* shape, could be added, but this is out of the scope of this paper.

Centroids with a higher score yield a more accurate mass value. For proof, a multi-compound standard with 199 substances ranging from *m*/*z* 103 to 916 was measured in ESI-positive mode by HPLC-HRMS. The concentration of each substance was 1 $\mathrm{\mu g}\cdot {\mathrm{L}}^{-1}$. In total, we considered 199 extracted ion chromatograms (EICs) using a 3-ppm window around the exact masses of the ${\left[M+H\right]}^{+}$ ion. This mass extraction window was chosen based on internal experiences with the expected mass accuracy of the applied HRMS instrument. For the masses in the EICs, the individual signal mass accuracy is determined by [29]:

14 $$\text{mass accuracy [ppm]}=\frac{m/{z}_{m}-m/{z}_{t}}{m/{z}_{t}}\times {10}^{6}$$

with $m/{z}_{m}$ being the measured mass and $m/{z}_{t}$ the exact mass of the ion.

**Fig. 3:**
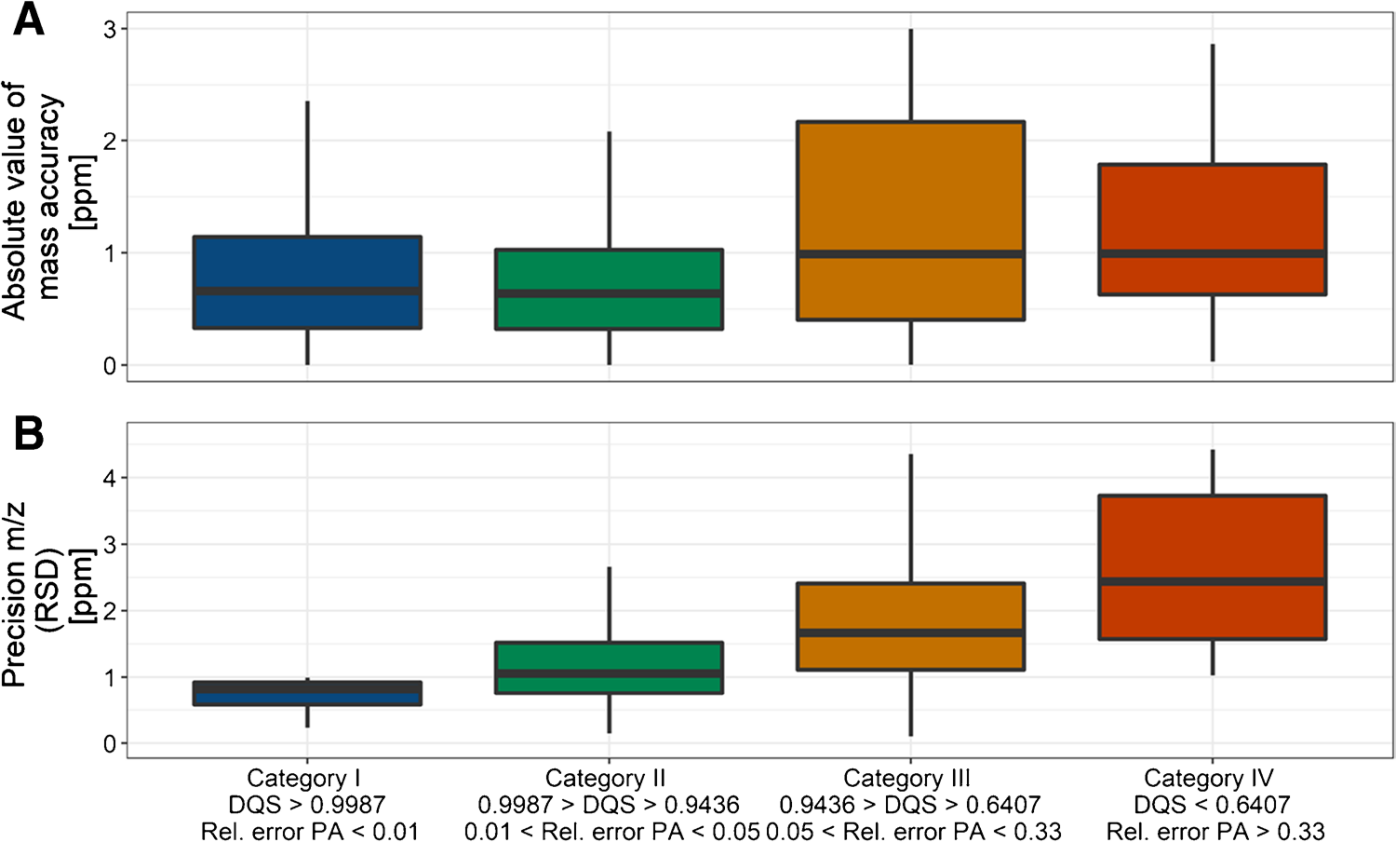
**A** Absolute mass accuracy in ppm compared for centroids falling into the categories I–IV. Centroids with a low DQS tend to show a higher deviation from the expected exact mass. **B** Precision in *m*/*z* compared for centroids falling into the categories I–IV given as relative standard deviation (RSD) in ppm. Centroids with a low DQS have lower precision in their *m*/*z* values. The categories are selected using the relative error for *Gaussian* peak area (1%, 5%, and 33%) and their corresponding DQS. The boxes enclose the interquartile range (IQR) and the median (horizontal line). The whiskers describe the quartiles $\pm$ 1.5 IQR

The boxplot in Fig. 3A shows the distribution of the absolute values of mass accuracies in ppm for the investigated standard substances for four categories (I–IV) covering different DQS ranges. A table that summarizes the categories can be found in the supplementary information (Table S5). The DQS ranges are chosen based on the relative error in the peak profile area (1%, 5%, and 33%) and are in accordance with the levels commonly used for significance testing.

It can be obtained from Fig. 3A that the median of the absolute value of mass accuracy is smaller in the case of high DQS values (categories I and II) than in the case of low DQS values (categories III and IV). Furthermore, the dispersion of the values, expressed by the interquartile range, is smaller with a higher DQS. Therefore, there is an indication that centroids with a high DQS are closer to their exact mass. It must be said here that the DQS is not a sharp criterion in this case, but also, some centroids with high DQS have a comparatively large mass error. However, a centroid with a low DQS is more likely to have a large mass error than a centroid with a high DQS.

Furthermore, centroids with low DQS have lower precision, i.e., increased *m*/*z* scattering over time. Studies on the precision in *m*/*z* can be performed in HRMS, e.g., by multiple direct injections of analyte solutions or by detecting the same ion species in a sequence of consecutive scans due to chromatographic separation. Figure 3B shows how the DQS is affected by the *m*/*z* precision. This experiment identified ion species in the HPLC-HRMS measurements that had fewer than three breaks in successive scans and a minimum size of 10 data points per group. For these groups, the relative standard deviation in *m*/*z* is determined to measure precision.

In addition to the higher probability that centroids with low DQS exhibit lower mass accuracy, Fig. 3B also indicates larger random errors. Correct assignment of mass to a molecular formula by accurate mass determination using HRMS, however, depends on mass accuracy and measurement precision [29, 30]. Therefore, for identifying unknown analytes based on the accurate mass determination, e.g., in the case of non-target screening, the interpretation of features with a high DQS should be better than results with low DQS as they show lower accuracy and precision. Processing peak profiles to obtain centroids goes along with an additional error source besides mass calibration and random error that affects the observed deviation between the exact and measured mass. For a peak with high quality (e.g., category I or II), centroiding can be performed without a significant loss of information because the shape is very close to the ideal model. On the other hand, non-ideal peaks have low qualities (i.e., category III or IV), making centroiding a lossy processing step, including information loss. However, the DQS indicates these cases by their value.

Following this aspect, centroid positions with an increased deviation in the *m*/*z* domain should be connected to decreased DQS values. This is particularly relevant for non-target screening by HPLC-HRMS, where the lack of reference substances means that it is not known in advance which masses form an EIC. The established feature detection algorithms could erroneously divide a related EIC based on this reason. There is an indication that an increased mass deviation prevents assignment to a group of masses, e.g., detecting regions of interest (ROIs) in *xcms’ centwave* algorithm [17] for binning. This is presented in a quantile–quantile plot (QQ plot) comparing the distribution of DQS values within and outside the obtained ROIs in the supplementary material in Fig. S4.

**Fig. 4:**
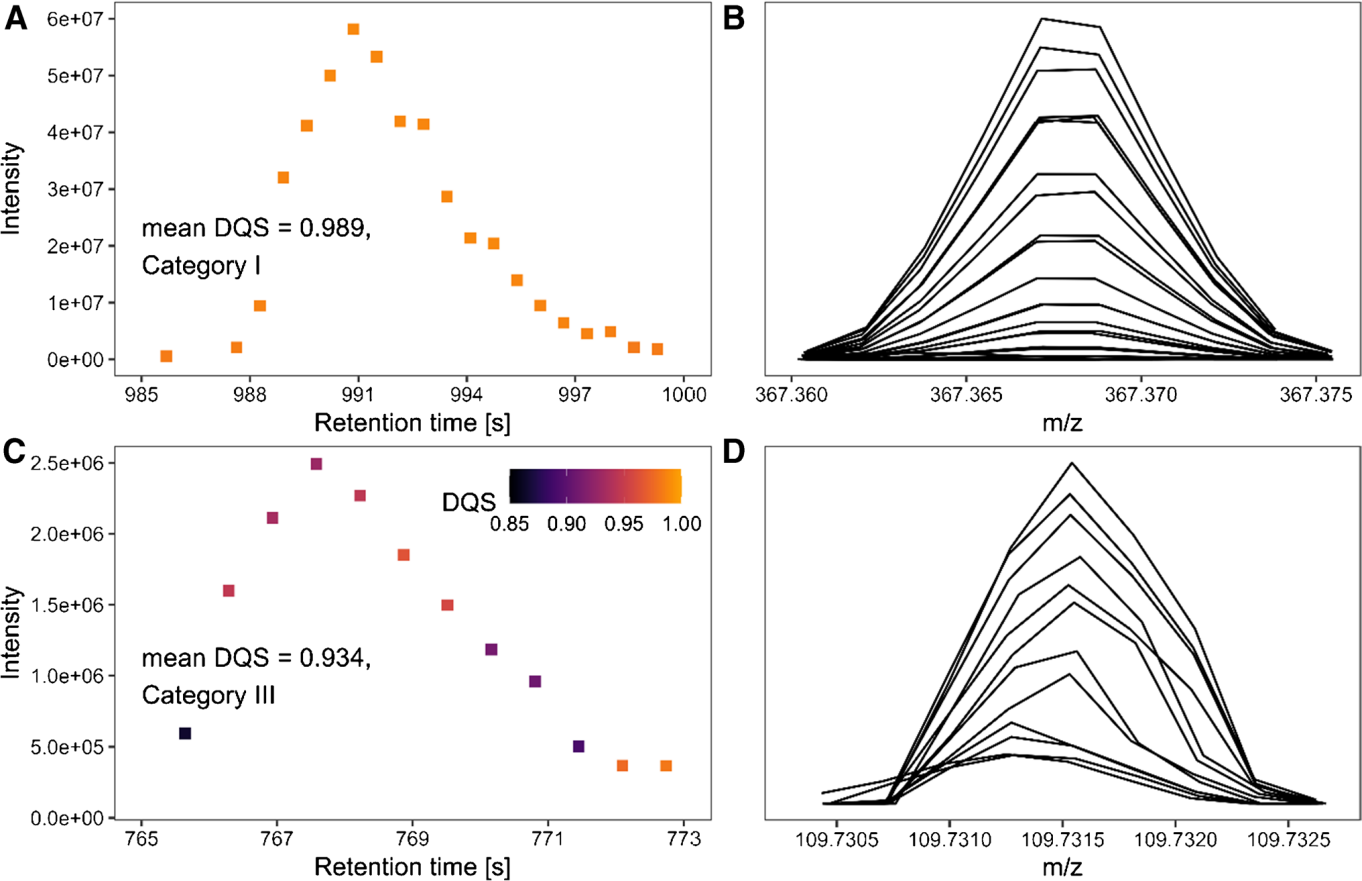
**A** Exemplary chromatographic profile that shows a pronounced peak. The Data Quality Score (DQS) of the centroids that formed this chromatographic peak is high (category I from Fig. 3), which indicates that the former peak profiles were of *Gaussian* shape. The color scale is given in subfigure **C**. **B** Peak profiles of the centroids applied in subfigure **A**. This subfigure shares the *y*-axis with subfigure **A**. The peak profiles are of *Gaussian* shape and symmetric; thus, the chance for the presence of non-resolved isobaric peaks or isotopic fine structures is reduced. **C** Chromatographic peak with lower DQS value (category II) in the data points. **D** The peak profiles of the centroids presented in subfigure **C** show higher asymmetry and lower match with the *Gaussian* model. Therefore, there is high potential for the presence of non-resolved underlying peaks in the peak profiles. This subfigure shares the *y*-axis with subfigure **C**

The DQS significantly benefits data prioritization usually performed within non-target screening (NTS). Prioritization in NTS helps highlight promising chromatographic peaks in extensive datasets to examine them in more detail and identify the analytes. Figure 4A and C show two chromatographic peaks that differ in the DQS values of the centroids that form these peaks. Figure 4B and D show the respective HRMS peak profiles of the centroids used for the chromatograms in Fig. 4A and C.

Without considering the DQS, both chromatographic peaks (Fig. 4A and C) are seen as equally reliable. This is because each chromatographic peak has an acceptable shape, s/n, assigned retention time, peak area, and average mass. However, DQS is sensitive to unresolved or interfering data in the *m*/*z* domain and, therefore, indicates whether the individual centroids are disturbed somehow, e.g., signal superposition. Therefore, the chromatogram in Fig. 4A should be prioritized. Due to the non-ideal nature of low-quality centroids having lower precision, accuracy, and a higher tendency to being not monoisotopic, these chromatographic peaks should not be assigned to an elemental composition or a specific substance. This aspect is not considered in conventional processing algorithms that analyze centroid data since the information about the original data quality is lost during centroid processing. We thus provide additional benefit with the development of the DQS for prioritization, as the user can detect and, if desired, sort out chromatographic peaks arising from centroids with low DQS.

Through fitting and Eq. 4, we can determine the peak profile width $\widehat{\upsigma }$. The peak width is of interest due to its meaning in mass resolution [31]:

15 $${\text{R}}=\frac{m}{\Delta m}$$

Usually, the mass resolution is expressed as a function of mass $m$ and peak width $\Delta \widehat{m}$ in FWHM instead of $\widehat{\sigma }$. For a *Gaussian* peak, the width $\Delta \widehat{m}$ in FWHM and the width $\widehat{\sigma }$ are interchangeable with the following relation:

16 $$\Delta \widehat{m}\text{ [FWHM]}=2\sqrt{2\,\mathrm{ln}\,2}\cdot \widehat{\sigma }$$

**Fig. 5:**
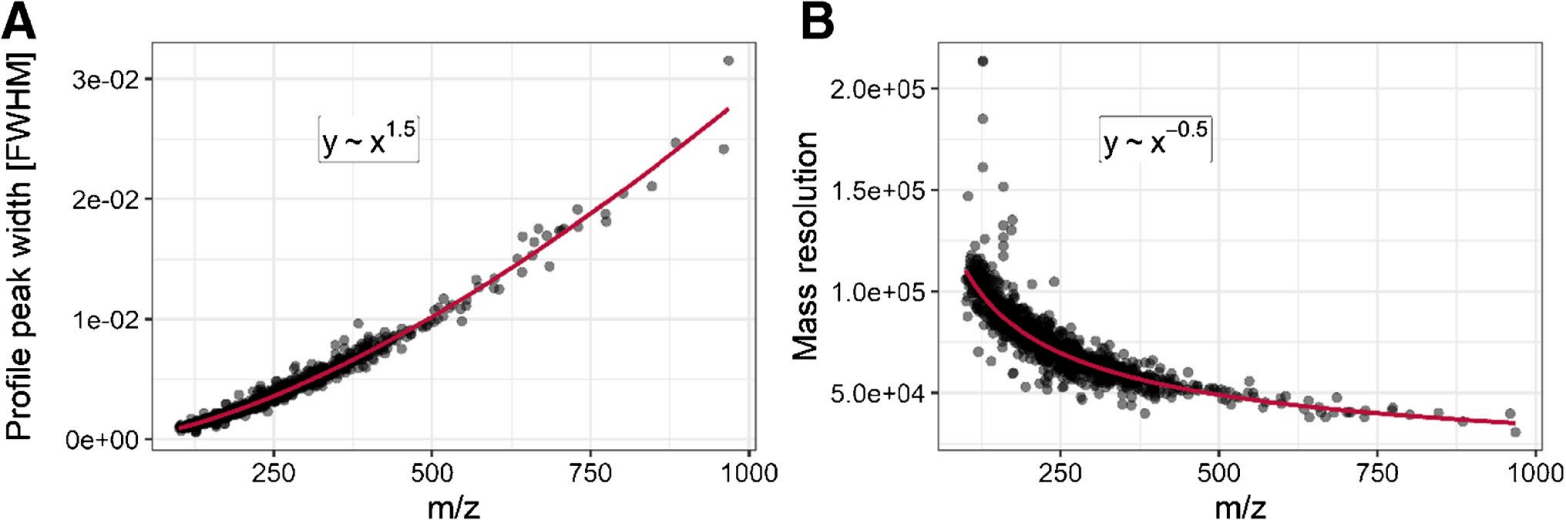
**A** Relationship between profile peak width [FWHM] and *m*/*z* centroid position. **B** Relationship between mass resolution and *m*/*z* centroid position. The mass resolution is calculated with Eqs. 15 and 16. To determine the relationship between the dependent and independent variables, a power law was fitted with non-linear regression

A mathematical derivation for that relation can be found in a specific section in the supplementary material. Figure 5A shows the functional relationship between profile peak width [FWHM] and *m*/*z*.

As shown, the peaks become broader with an increase in *m*/*z*. Figure 5B plots the same data, but peak width is replaced by mass resolution. Mass resolution is calculated via Eqs. 15 and 16, respectively. The fit function in Fig. 5B reveals the functional relationship mass resolution ${\text{R}}\sim m/{z}^{-0.5}$ which is commonly observed in Orbitrap-MS [32]. The functional relation between mass resolution and *m*/*z* depends on the MS instrument type; e.g., in the case of TOF–MS, it is linear [33]. It can be obtained that the resolution is not a fixed but a distributed value for a certain mass. This phenomenon can be based on the superposition of two non-resolved mass peaks, which decreases the effective mass resolution. Furthermore, there is a finite and very small number of data points per peak. Therefore, even a change of $\pm$ 1 data point can have a considerable influence on the peak width. The fitted mass resolution in Fig. 5B approximates 78,000 at *m*/*z* 200 with a prediction error of $\pm$ 17,800 (*α* = 0.05) which is in agreement with the instrumental resolution of 70,000 described in the experimental section. Thus, our algorithm robustly gives information on the mass resolution of each peak on a certain *m*/*z* value.

The quantitative description of a peak can be given either by the peak height or the peak area. Most algorithms, e.g., *msConvert*, specify the height. However, due to our centroiding workflow, we can also determine the peak area using Eq. 6. As explained above, the peak width (or mass resolution) depends on *m*/*z*. This results in mass peaks at high *m*/*z* having a low height under the assumption of a constant area. For identical or very similar *m*/*z* values, the effect of change in mass resolution may be neglected. Due to the described resolution-related effect of the emergence of broader but lower peaks in high mass ranges, discrimination effects for these broad peaks can be observed. This can occur when peak height-based intensity thresholds are used within the usual data processing routines. These discrimination effects are reduced for high masses by stating the area as the quantifier for peak intensity. However, the peak area is more prone to be affected by non-resolved, low-quality peaks, which can be easily solved by filtering out these peaks with our presented DQS. Thus, we state both peak height and peak area to give the user a choice for one of the two quantifiers.

### Validation of centroiding with msConvert

When comparing our results with *msConvert*, the first question is whether differences in the peak detection have occurred. For this, the filter criteria (${\widehat{\beta}}_{2}<0$ and at least four data points) are neglected at first. Both our algorithm and *msConvert* detect 3,602,151 profile peaks for our example data file. Manual inspection of the peaks showed that the integration limits were set identically. This suggests that the two algorithms consider the same peak profiles for centroiding.

However, the Thermo-specific centroiding algorithm implemented in *msConvert* also centroids peaks with ≤ 3 data points. As we have established filter criteria in the section above, these detected profile peaks are not in our centroid list. For the example data file analyzed during this study, 1.38% of the detected peaks did not fulfill both mentioned requirements to obtain a meaningful peak fit (criterion 1: 1.39%, criterion 2: 1.38%). It is questionable that peaks with two data points can be determined with sufficient statistical confidence. As our concept aims to characterize data quality to reduce the chance of recording false positives in mass spectra, we do not further consider these peaks.

For the remaining pairs, the peak height of our algorithm was compared with the intensity of *msConvert*. A graphical representation can be found in the supplementary material in Figure S7. There is a linear relationship between our peak height and the intensity of *msConvert* with a coefficient of determination of 1.000. Furthermore, the slope of linear regression is slightly greater than 1, which means that our peak heights are consistently smaller compared to *msConvert*. However, through the very good correlation, the comparability of the results in terms of intensity is assured.

**Fig. 6:**
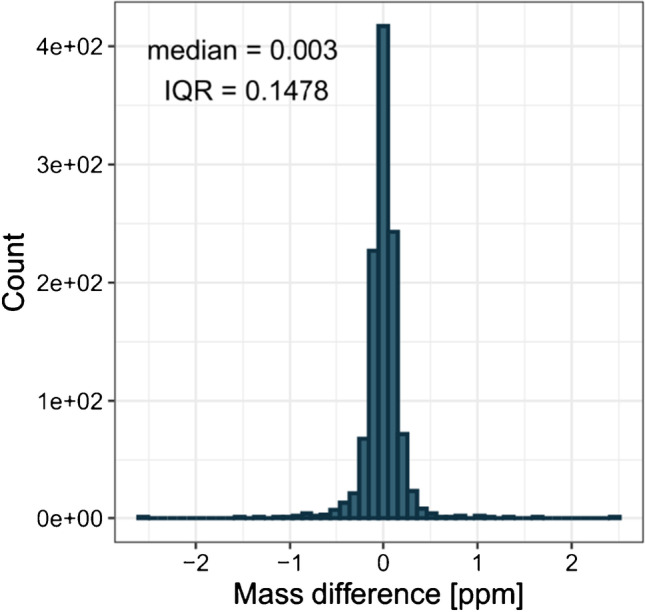
Histogram with 50 classes of relative *m*/*z* difference [ppm] between our obtained centroids and *msConvert*’s results. Median and interquartile range (IQR) are determined from 1129 difference values

The second aspect that must be compared is the centroid position. To do so, the relative difference in parts per million between our centroids and *msConvert* is calculated. The results are plotted as a histogram in Fig. 6.

The ppm difference is distributed around a median of 0.003 with an interquartile range of 0.1478. The ppm deviation was chosen because it is less dependent on the magnitude of *m*/*z* than the absolute deviation (e.g., in mDa). The ppm difference is close to zero. The maximum absolute deviation does not exceed 2.6 ppm. The observed ppm deviation is acceptable because mass resolution precision errors are 4 × smaller than expected. Furthermore, identifying the analytes and determination of their elemental composition based on the accurate mass measurement should not be constrained by this difference [34–36].

The results indicate that our algorithm is fully comparable to the results of the Thermo centroiding algorithm provided in *msConvert* but can additionally reflect information about the data quality.

### Performance analysis of algorithm

We developed the algorithm in different programming languages (R, Python, and Julia) to increase accessibility. It is important to ensure whether all developed tools provide the same results. In this context, Myers et al. (2017) showed that the *centwave* algorithm that is implemented in the R packages *xcms* and *MZmine 2* detects different features [37].

No differences were found between the processing results of the different programming languages for centroid position, intensity, or DQS. The second performance aspect is the processing time spent by the algorithm. To clarify the challenge faced by algorithms using second-order linear regression: the centroiding workflow groups 50,000,000 data points into 3,500,000 peak profiles which subsequently are converted into 3,500,000 centroids. Through our centroiding algorithm, the data file is reduced from 1064 to 439 MB.

A central parameter in the comparison of the different programming environments is the computing speed. The speed comparison was performed using a LENOVO ThinkPad P50-20EQS4QL11 with an Intel Core i7-6820HQ (4 × 2.7 GHz) processor, 16 GB DDR4 RAM (2 × 8 GB), 512 GB SSD M.2. The algorithm is executed fastest with Julia (60 s), followed by Python (105 s) and R (155 s). As a benchmark, *msConvert* took 55 s to convert profile *.mzXML to centroid *.mzXML. Julia is known for its high performance. However, Python and R can also solve the challenge in a reasonable amount of time. R is the slowest programming language but is widely used in the analytical chemistry community. Many processing and analysis tools for HRMS are implemented in R, making our algorithm convenient to couple with the other tools. However, even the slowest version of the algorithm implemented in R performs approximately 23,000 regressions per second, which is considerably fast. It must be mentioned here that no interface to other programming languages, such as C + + in the case of R, was used to speed up the computations. The efficiency of the code in R and Python is made possible primarily by using sparse matrix algebra.

## Conclusion

The DQS is suitable to describe the quality of centroids originating from profile mass spectra. The DQS enables error propagation for centroiding to filter out low-quality, highly uncertain centroids, which substantially facilitates data interpretation. For Orbitrap-MS, peak profiles are mostly *Gaussian*-shaped [14]. The DQS is appended to the conventional centroid list as it allows detecting low-precision, low-accuracy centroids and can be applied for feature detection in HPLC-HRMS. Additionally, our algorithm efficiently determines the peak profile widths and associates them with their centroids. Estimating peak widths is of interest for subsequent data processing steps, e.g., to decide how the *m/z* domain should be grouped for feature detection. Our algorithm is significantly improved compared to conventional centroiding algorithms in which the central information (quality, width) on the former peak profile quality is lost.

## Supplementary Information

Below is the link to the electronic supplementary material.

Supplementary file1 (PDF 2243 KB)

Supplementary file2 (JL 12 KB)

Supplementary file3 (R 20 KB)

Supplementary file4 (PY 7 KB)
