# Supplementary material for: Development of a scoring parameter to characterize data quality of centroids in high-resolution mass spectra
Source: Anal Bioanal Chem. 2022 Jul 25;414(22):6635–45. doi: 10.1007/s00216-022-04224-y (PMC9411079; doi:10.1007/s00216-022-04224-y)
Supplement: Supplementary file 1 — Supplementary file1 (PDF 2243 KB) [file 216_2022_4224_MOESM1_ESM.pdf]

Supplementary Information: Development of a Scoring Parameter to Characterize Data Quality of Centroids in High-Resolution Mass Spectra.

Max Reuschenbach[1], Lotta L. Hohrenk-Danzouma[1,2], Torsten C. Schmidt[1,2,3] and Gerrit Renner[1,2*]

[1]Instrumental Analytical Chemistry, University of Duisburg-Essen, Universitätsstr. 5, 45141 Essen, Germany.
[2]Centre for Water and Environmental Research (ZWU), University of Duisburg-Essen, Universitätsstr. 2, 45141 Essen, Germany.
[3]IWW Water Center, Moritzstr. 26, 45476 Mülheim an der Ruhr, Germany.

*Corresponding author(s). E-mail(s): gerrit.renner@uni-due.de;

1 Sampling, sample preparation and instrumental analysis

To validate the developed centroiding algorithm examples samples were processed. The samples measured were grab samples taken at the effluent of the wastewater treatment plant in Warburg (Stadtwerke Warburg GmbH, Warburg, Germany). Details can be found in Itzel et al. (2020).[1]
A solid phase extraction was performed for sample preparation within 48 h after sampling. The cartridges (150 mg, 6 mL, Oasis HLB, Waters, Eschborn, Germany) were conditioned with 2 x 5 mL methanol and equilibrated with 2 x 5 ml water. After drying the cartridges under vacuum, the loaded cartridges were stored at -18 °C until further analysis. 5 x 5 mL MTBE were used for elution.

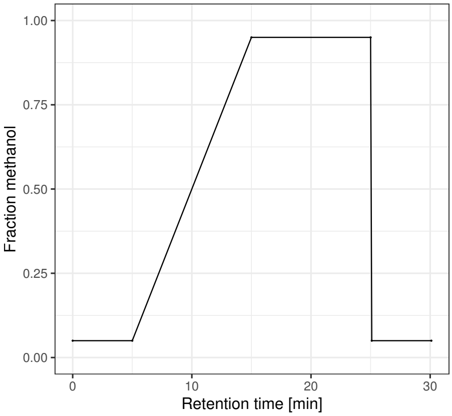

Fig. S 1 Solvent gradient for the analysis of wastewater grab samples with HPLC-HRMS. The solvents were a) water + 0.1 % formic acid and b) methanol + 0.1 % formic acid. The y-axis presents the fraction of the organic solvent methanol during the gradient program.

After elution, the samples were measured within 48 h. The liquid chromatography was performed using a Dionex UltiMate 3000 HPLC system (Thermo Scientific, Bremen, Germany) with a gradient method on a XSelect HSS T3 (2.1 mm x 75 mm, 3.5 µm particle size, Waters, Milford, MA, USA). The mobile phase was consisting of eluent A: LC-MS grade water (Th. Geyer GmbH & Co. KG, Renningen, Germany) + 0.1 % formic acid (99 % purity, VWR Chemicals, Darmstadt, Germany), and eluent B: methanol (LC-MS grade, Th. Geyer GmbH & Co. KG, Renningen, Germany) + 0.1 % formic acid. The eluent gradient is displayed in figure S1. The injection volume was 20 µL and the flow rate 0.35 mL/min. The ion source was a heated electrospray ionization which operation parameters are specified in table S1. The scanning settings of the Orbitrap mass spectrometer are given in table S2. MS2 Spectra

were not included in this data evaluation, but, however, influence instrument cycle time.

Table S 1 Parameter of heated electrospray ionization (HESI).

Paramter	Specification
Polarity	positive/negative
Sheath gas flow rate	37
Aux gas flow rate	15
Sweep gas flow rate	1
Spray voltage	3.5
Capillary temperature	320
S-Lens RF level	50
Aux gas heater temperature	50

Table S 2 Orbitrap-MS scanning setting for Full MS and data-dependent MS2 measurements (ddMS2).

	Full Scan	ddMS2
Resolution	70'000	17'500
AGC Target	1E06	5E04
Max. injection time	100 ms	50 ms
Scan range	m/z 100-1000	-
Loop count	-	-
Isolation window	-	1.4 m/z
NCE	-	30,60
Intensity threshold	-	2E03

2 Data handling

2.1 Applied software packages from third parties

The algorithms are implemented in the three programming environments R, Julia and Python and are depending on packages of third parties. The following packages have been loaded and applied in the programming code (see table S3).

Table S 3 Third party software packages applied

Programming language	Packages
R	Matrix, mzR, R.utils, pracma, data.table, tidyverse
Julia	MzXML
Python	Pyteomics

2.2 xcms Parameter

For processing of the data file with *xcms* the parameter from table S4 were chosen. All other parameter were kept in the default settings. The processing was performed in R using the package xcms.

Table S 4 Parameter set for processing of HPLC-HRMS data with xcms

Parameter	Specification
method	centwave
ppm	3
snthresh	10
peakwidth	10-60
mslevel	1
Noise	0

3 Derivation of the Data Quality Score

3.1 Orbitrap HRMS peak profiles

The error $\Delta\hat{A}$ in the Gaussian peak $\hat{A}$ area is calculated with the following set of equations that is based on the propagation of errors:

$$k_1 = \exp\left(2\hat{\beta}_0 - \frac{\hat{\beta}_1^2}{2\hat{\beta}_2}\right) \tag{1}$$

$$k_2 = \sqrt{2\pi}\exp\left(\hat{\beta}_0 - \frac{\hat{\beta}_1^2}{4\hat{\beta}_2}\right) \tag{2}$$

$$k_3 = \sqrt{\frac{1}{2\hat{\beta}_2}} \tag{3}$$

$$d_0 = \frac{\pi k_1}{\hat{\beta}_2}\Delta\beta_0^2 \tag{4}$$

$$d_1 = \frac{\pi\hat{\beta}_1^2 k_1}{4\hat{\beta}_2^3}\Delta\hat{\beta}_1^2 \tag{5}$$

$$d_2 = \frac{k_2}{4\hat{\beta}_2{}^2 k_3} - \frac{k_2\hat{\beta}_1^2 k_3}{4\hat{\beta}_2^2}^2 \Delta\hat{\beta}_2^2 \tag{6}$$

$$\Delta A = \sqrt{d_0 + d_1 + d_2} \tag{7}$$

with $\hat{\beta}_0$, $\hat{\beta}_1$, $\hat{\beta}_2$ being the parameter of weighted second-order linear regression and $\Delta\hat{\beta}_0$, $\Delta\hat{\beta}_1$, $\Delta\hat{\beta}_2$ their respective standard errors. The Data Quality Score (DQS) introduced in the publication is estimated by the relative error of the peak area. The functional relationship between the two quantities is shown by the equation 8 and the figure S2.

$$\text{DQS} = 1 - \text{erf}\left(\frac{\Delta\hat{A}}{\hat{A}}\right) \quad (8)$$

with $\Delta\hat{A}$ being the error in the *Gaussian* peak area, $\hat{A}$ being the *Gaussian* peak area. erf(x) is the *Gaussian* error function.

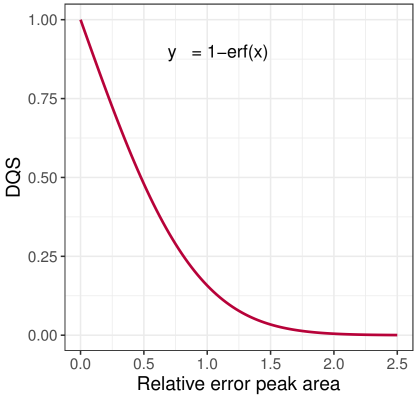

Fig. S 2 Plot of the function used to generate the Data Quality Score (DQS) from the relative error of the peak area.

To facilitate the selection of a DQS for the user, four qualitative categories are introduced in table S5.

Table S 5 DQS categories and their associated relative error in *Gaussian* peak area.

Category	Rel. error peak area [%]	DQS range
I	<1.0	>0.9887
II	>1.0 & <5.0	<0.9887 & >0.9436
III	>5.0 & <33.0	<0.943 & >0.6407
IV	>33.0	<0.6407

3.2 TOF HRMS peak profiles

The routine described above is limited to *Gaussian*-shaped peaks observed,e.g., in Orbitrap-MS. However, our algorithm can also be extended to match asymmetric peak profiles, e.g., TOF data. The peak model must be adapted to apply the concept of data quality estimation for these instruments, as the peak profiles are usually asymmetric. Therefore, we cannot use the *Gaussian* model as asymmetry is not considered. Thus, we decided to apply the *Bi-Gaussian* model that fits the left and the right half of a peak independently and, therefore, allows asymmetry:[2]

$$\hat{I}(x) = \begin{cases} \hat{I}_{01} \exp\left(\frac{-(x-\hat{x}_0)^2}{2\hat{\sigma}_1^2}\right), & \text{if } x \leq \hat{x}_0 \\ \hat{I}_{02} \exp\left(\frac{-(x-\hat{x}_0)^2}{2\hat{\sigma}_2^2}\right), & \text{if } x \geq \hat{x}_0 \end{cases} \tag{9}$$

with $\hat{I}_{01}$ and $\hat{I}_{02}$ left and right half intensity and $\hat{\sigma_1}$ and $\hat{\sigma_2}$ the left and right half-width. A big advantage of the *Bi-Gaussian* compared to other peak models considering asymmetry is that it can be linearized following the procedure from Caruana et al. (1986), making the algorithm computationally very efficient:[3]

$$\ln \hat{I}(x) = \begin{cases} \ln \hat{I}_{01} - \frac{1}{\hat{\sigma}_1^2} x_c^2, & \text{if } x \leq \hat{x}_0 \\ \ln \hat{I}_{02} - \frac{1}{\hat{\sigma}_2^2} x_c^2, & \text{if } x \geq \hat{x}_0 \end{cases} \tag{10}$$

Eq. 10 describes two halfs of a parabola of the form $y = a + bx_c^2$ with $x_c = x - \hat{x}_0$. Essentially, the procedure is analogous to symmetric peaks, except that there is a smaller number of parameters in the polynomial function, and the effect on the statistical degrees of freedom must be taken into account:

$$\hat{I}_{0i} = \exp(a_i) \tag{11}$$

$$\hat{\sigma}_i = \sqrt{-\frac{1}{2b_i}} \tag{12}$$

$$\hat{A}_i = \hat{I}_{0i} \ \hat{\sigma}_i \ \sqrt{2\pi} \tag{13}$$

$$\hat{A}_{tot} = \hat{A}_1 + \hat{A}_2 \tag{14}$$

Further, a asymmetry factor γ can be calculated:

$$\gamma = \frac{\hat{\sigma}_2}{\hat{\sigma}_1} \tag{15}$$

$$\Delta \hat{A}_i = \sqrt{\left(\frac{\partial \hat{A}_i}{\partial \hat{I}_{0i}}\right)^2 \Delta \hat{I}_{0i}^2 + \left(\frac{\partial A_i}{\partial \hat{\sigma}_i}\right)^2 \Delta \hat{\sigma}_i^2} \tag{16}$$

$$\Delta \hat{A}_i = \sqrt{\pi} \sqrt{\frac{(-4b_i \Delta a_i^2 - \Delta b_i^2) \exp(2a_i)}{b_i}} \tag{17}$$

$$DQS = 1 - \text{erf}\left(\frac{\Delta \hat{A}_1 + \Delta \hat{A}_2}{\hat{A}_{tot}}\right) \tag{18}$$

Two exemplary asymmetric peak profiles from TOF-MS, one with high and one with low DQS are given in figure S3.

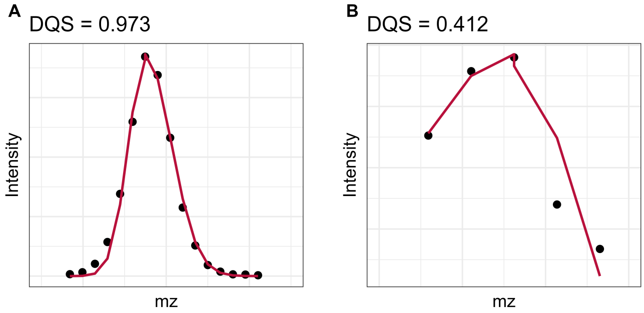

Fig. S 3 Two exemplary peak profiles from TOF-MS (asymmetry factor A: 1.54 and B: 0.52) fitted with the *Bi-Gaussian* model and their associated DQS.

4 Influence of low quality centroids on the construction of regions-of-interest (ROI) with xcms

In this context, we considered the wastewater dataset, centroided conventionally using *msConvert*, and used *xcms* for ROI calculation. the *xcms* parameter are given in table S4 Afterwards, all the centroids from our new algorithm were assigned to ROIs from the conventionally evaluated data. Those centroids that cannot be assigned to any ROI were defined as "outside". A quantile-quantile plot (QQ-plot) comparing the distribution of DQS values within and outside the obtained ROIs is given in figure S4.

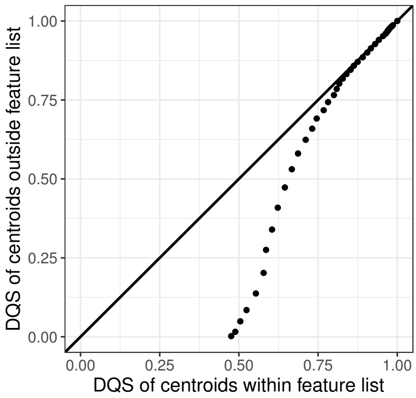

Fig. S 4 Quantile-quantile plot (QQ-plot) between the DQS of centroids that are within the feature list obtained by *xcms'* centwave algorithm (n=36'260) and outside the feature list (n=3'531'974). In a QQ-plot the quantiles of one variable are plotted against the quantiles of a second variable which allows to compare their distributions.

The QQ-plot shows a deviation from the black bisecting line, indicating different DQS distributions within and outside ROI. The lowest DQS within

the ROIs is 0.48. The quantile values do not coincide, resulting in a slope greater than one, indicating differences in data spread. Within the ROIs, centroids show an increased DQS. This could be since those centroids with low DQS could not be meaningfully assigned to an ROI due to their potentially high mass deviation. Lower DQS centroids tend towards the edge of the mass extraction window. Therefore, DQS can be considered to calculate ROIs' weighted average masses to improve mass accuracy compared to the conventional unweighted ROIs' average masses.

5 Simulating the effect of peak-to-peak resolution on Data Quality Score

In the following, it shall be analyzed how the Data Quality Score is affected by the presence of partially or non-resolved neighboring peak profiles in HRMS. Therefore, two *Gaussian* peaks, one left main peak and one right secondary peak are simulated according to the peak shape observed in Orbitrap-MS. The positions for main and secondary peak are $x_{0,1}$ and $x_{0,2}$. Thus, the peak-to-peak resolution is defined as:

$$R_p = 2\frac{x_{0,2} - x_{0,1}}{\sigma_1 + \sigma_2} \tag{19}$$

with $x_{0,2} > x_{0,1}$. Assuming $\sigma_1 = \sigma_2$, Equation 19 can be simplified to:

$$R_p = \frac{x_{0,2} - x_{0,1}}{\sigma_1} \tag{20}$$

Equal width σ_1 is assumed because both peaks originate from the nearly same m/z range. In a simulation, two *Gaussian* peaks (I and II) are generated with varying R_p. In principle, a differentiation can be made between two cases: Either the two peaks are separated, indicated by the formation of a local minimum between the peaks, or they are not separated. In the second case, the two peaks cannot be considered separately. The differentiation by means of local minima mentioned here is also implemented in this form in our algorithm. The height of the left peak (I) is set to 1, and the second peak height (II) is varied in the range of 0-1. Figure S5 shows the DQS of the peak I affected by R_p and the height of peak II. The number of data points per peak was adjusted on the experience from Orbitrap-MS, i.e., 7 points. The peaks are fitted independently when a local intensity minimum is observed in the sum of their signals in analogy with the study's method. Otherwise, their sum signal is considered as one peak. Simulation results ($n = 5000$) are presented in figure S5. No noise is incorporated in this simulation leading to higher DQS values than real measurement data.

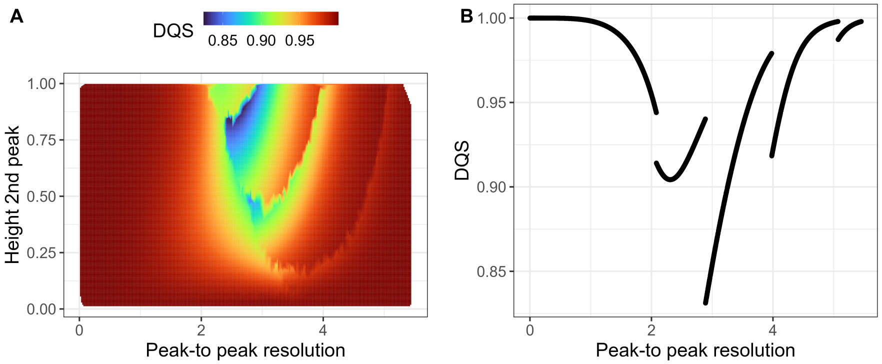

Fig. S 5 A: Simulation: the influence of peak to peak resolution and the height of the second peak on the Data Quality Score ($n = 5000$). The height of the main peak is set to 1, while the height of the secondary peak is varied in the interval [0,1]. B: Relation between DQS and peak-to-peak resolution for a secondary peak of height 1. As seen in subfigures A and B, the DQS increases with increase in peak-to-peak resolution in a step-wise manner. This can be attributed to a change in the statistical degrees of freedom on which the DQS depends the better both peaks are separated.

For peaks with the same position ($R_p = 0$), the simulation results in DQS values close to 1 independent of the second peak height as the sum signal has an ideal shape and, therefore, a high DQS. If the position and height of the second peak are altered, the DQS is affected: At first, the DQS decreases slowly with distance as a distortion in peak shape is observed. At a critical distance a new local minimum appears that is now used to split the peaks. As a result, the statistical degrees of freedom of peak I are decreased drastically. At the same time peak distortion is increased, so that the deviation from the *Gaussian* model is increased. This leads to abruptly lowered DQS. After a global minimum the DQS increases with R_p due to the fact that the mutual influence of the peaks is reduced due to reduced peak overlap. The model fit becomes better, but the statistical degrees of freedom are also increased, so that the resulting DQS tends to approach 1 in a step-wise manner. This step-wise manner is shown for a secondary of peak height 1 in figure S5B. For high R_p values DQS values close to 1 are observed as the *Gaussian* peaks do not affect each other. Overall, the higher the intensity of the secondary peak the stronger is the distortion and the lower the DQS of peak I.
Through simulation, it can be shown that our DQS is suitable to estimate the quality of the peak profiles, since the influence of non-resolved neighboring peaks, e.g., isotopic fine structures or isobaric analytes, onto centroid reliability can be quantified.

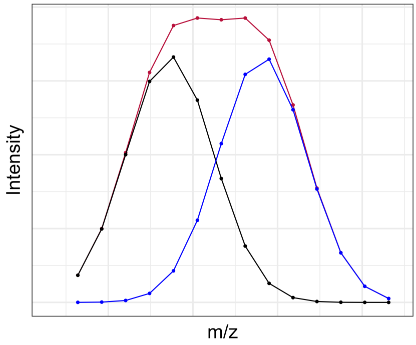

Fig. S 6 Critical resolution ($R_p = 2.12$) for two equal height *Gaussian* peaks at which the sum signal of both peaks develop a local minimum. This local minimum was used to split profile peaks that are not fully separated in the simulation in figure S5.

6 Validation of centroiding with msConvert

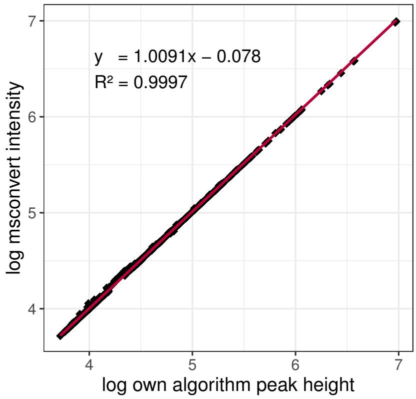

Fig. S 7 Correlation between log *msConvert* intensities and the log peak height gathered by our own algorithm's peak height (n=1'129).

7 Relationship between full-width half-maximum (FWHM) and standard deviation of the Gaussian peak

To determine the FWHM, the task is to find the position x_h where the intensity is half the maximum intensity. The *Gaussian* peak can be described by the following function.

$$\hat{I}(x) = \hat{I}_0 \cdot \exp\left(-\frac{(x - \hat{x}_0)^2}{2\hat{\sigma}^2}\right) \tag{21}$$

The intensity is maximal at the center of the *Gaussian* peak $\hat{x}_0$.

$$\frac{1}{2}\cdot\hat{I}(\hat{x}_0)=\frac{1}{2}\hat{I}_0\cdot\exp\left(-\frac{(\hat{x}_0-\hat{x}_0)^2}{2\hat{\sigma}^2}\right)=\frac{1}{2}\hat{I}_0 \tag{22}$$

Thus, the intensity at the position x_h is:

$$\hat{I}(x_h)=\frac{1}{2}\hat{I}_0=\hat{I}_0\cdot\exp\left(-\frac{(x_h-\hat{x}_0)^2}{2\hat{\sigma}^2}\right) \tag{23}$$

Simplifying and transforming the equations yields:

$$\frac{1}{2}=2^{-1}=\exp\left(-\frac{(x_h-\hat{x}_0)^2}{2\hat{\sigma}^2}\right) \tag{24}$$

$$-\ln 2=-\frac{(x_h-\hat{x}_0)^2}{2\hat{\sigma}^2} \tag{25}$$

$$2\ln 2\hat{\sigma}^2=(x_h-\hat{x}_0)^2 \tag{26}$$

$$\sqrt{2\ln 2\hat{\sigma}^2}=\sqrt{2\ln 2}\hat{\sigma}=x_h-\hat{x}_0 \tag{27}$$

Due to the fact that the *Gaussian* peak is symmetric FWHM follows:

$$\text{FWHM}=2\ |(x_h-\hat{x}_0)|=2\sqrt{2\ln 2\hat{\sigma}^2}\approx 2.3548\cdot\hat{\sigma} \tag{28}$$

References

[1] Itzel F, Baetz N, Hohrenk LL, Gehrmann L, Antakyali D, Schmidt TC, Tuerk J. Evaluation of a biological post-treatment after full-scale ozonation at a municipal wastewater treatment plant. Water Res 2020;170:115316.

[2] Buys T, De Clerk K. Bi-Gaussian fitting of skewed peaks. Anal Chem 1972;44(7):1273–1275.

[3] Caruana RA, Searle RB, Heller T, Shupack SI. Fast algorithm for the resolution of spectra. Anal Chem 1986;58(6):1162–1167.
